# Genetic Diversity of the root‐knot nematode *Meloidogyne enterolobii* in Mulberry Based on the Mitochondrial COI Gene

**DOI:** 10.1002/ece3.6282

**Published:** 2020-06-01

**Authors:** Hudie Shao, Pan Zhang, Chunping You, Chuanren Li, Yan Feng, Zhenwen Xie

**Affiliations:** ^1^ College of Agriculture Yangtze University Jingzhou China; ^2^ The innovative Institute for plant health Zhongkai University of Agriculture and Engineering Guangzhou China

**Keywords:** COI mitochondrial, Genetic diversity, *Meloidogyne enterolobii*, Mulberry

## Abstract

This study explores the genetic diversity and structure of *Meloidogyne enterolobii* in mulberry in China. The COI mitochondrial gene (mtCOI) in *M.enterolobii* populations in Guangdong, Guangxi, and Hunan Provinces was PCR‐amplified, sequenced, and analyzed for genetic diversity. The total number of variations, haplotypes (Hap), the average number of nucleotide differences (k), haplotype diversity (H), and nucleotide diversity (π) of mtCOI were 25, 11, 4.248, 0.900, and 0.00596, respectively. Insignificant differences in Fst value (0.0169) and a high level of gene flow (7.02) were detected among the 19‐mulberry root‐knot nematode populations, and high genetic variation within each population and a small genetic distance among populations were observed. Both phylogenetic analyses and network mapping of the 11 haplotypes revealed a dispersed distribution pattern of 19 mulberry root‐knot nematode populations and an absence of branches strictly corresponding to the 19 range sampling sites. The neutrality test and mismatch analysis indicated that mulberry root‐knot nematode populations experienced a population expansion in the past. The analysis of molecular variance (AMOVA) revealed that the genetic differentiation of *M. enterolobii* was mainly contributed by the variation within each group. No significant correlation was found between the genetic distance and geographical distance of *M. enterolobii* populations. The findings of this study provide a profound understanding of the *M. enterolobii* population and will inform the development of strategies to combat and manage root‐knot nematodes in mulberry.

## Graphical abstract

This study explores the genetic diversity and structure of Meloidogdyne enterolobii (M. enterolobii) in mulberry in China. The mitochondrial COI gene (mtCOI) in mulberry root‐knot nematode populations in Guangdong, Guangxi, and Hunan Provinces was PCR‐amplified, sequenced, and analyzed for genetic diversity.

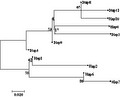

## 1 INTRODUCTION

*Meloidogyne enterolobii* is a root‐knot nematode that causes serious damage to economic and food crops (Chen, Wan, & Chen, 2016). It has become one of the most threatening pathogenic nematodes in both tropical and subtropical regions of the world, with estimated potential yield loss of 20% (Zhuo et al., 2008). *Meloidogyne enterolobii* was first discovered on *Euterolobium coutortisiliquum* in Zhangzhou, Hainan Province, China (Yang & Eisenback, 1983). *Meloidogyne enterolobii* has a strong pathogenicity and a wide host range (Wang, Rui, & Fu, 2015). In recent years, *M. enterolobii* had gradually spread from the south to the north of China (Wu, Zhang, & Xue, 2019). It has also been found in Africa, America, and Europe (Onkendi et al., 2013).

Ribosomal DNA (rDNA) has been used as a molecular marker to identify and phylogenetically characterize different nematodes by recent studies. For example, the rDNA‐PCR technology has been employed to identify *Bursaphelenchus* spp. (Jiang, Liang, & Zhen, 2005) and Pratylenchus spp. (Mizukubo, Sugimura, & Uesugi, 2007). Mitochondrial DNA (mtDNA) has emerged as another useful tool for the genetic and taxonomic studies of various parasitic nematodes due to its small molecular weight, simple and stable structure, and relatively conserved gene composition (Duan, 2013). For example, Sun, Liao, and Li (2005) used the mitochondrial COII‐LrRNA gene fragment to distinguish between different Meloidogyne spp. populations. Wang (2015) determined the genetic diversity of the mitochondrial mtCOI gene in 318 soybean cyst nematode individuals from 16 populations in China. The results showed that there was a certain genetic differentiation among various groups, but the level of gene exchange was also high. Tu, Gao, and Zhou (2007) used the COI gene to study the genetic diversity of 6 local chicken breeds in China. The results showed that there were 22 mutation sites in the gene sequence, and the COI gene sequence of the 6 local chicken breeds had high genetic diversity. It has been reported already using GBS method by Rashidifard et al. (2018) and AFLP, ISSR, and RAPD methods by Tiago, Siqueira, and Castagnone‐Sereno (2010), but they have not used COI mtDNA. Thus, this research aims to explore the structure and genetic differentiation of *M. enterolobii* populations by integrating bioinformatic and the DNA barcoding technologies. Therefore, this research, by applying PCR and DNA bar codes techniques, combining with means of bioinformatics analyzes and compares population genetics indexes of *M. enterolobii*, and lays the foundation for mulberry breeding and the prevention and cure of *M. enterolobii* disease in the future.

## 2 MATERIALS AND METHODS

### 2.1 Nematode collection

The nematode samples used in this experiment were collected from the main sericulture areas in Guangdong, Guangxi, and Hunan Provinces, China (Table 1).

**Table 1:** 19 *Meloidogyne enterolobii* samples from mulberry logicalities in china that has been used during this study

Group	City	town	Shorthand	Variety	Acquisition time	number of soil samples(Female/ Article)	Strain number
YZ	Guangzhou	Baiyun	GB	Yuexiu63	2016.7; 2017.8	10	GB 1
	Huadu	Baosang	HB	Japanese mulberry	2016.5; 2017.10	10	HB2
	Guangzhou	Huanong	GH	Shi 4	2018.4	10	GH 3
	Yunfo	Luoding	YL	Tang10 × 109	2017.9	10	YL 4
	Foshan	Shede	FS	Tang10	2017.8	10	FS5
	Guangzhou	Nansha	GN	Sijiguo sang	2018.5	10	GN 6
	Guangzhou	Pan	GP	Da10	2018.5	10	GP 7
YB	Yingde	Dawan	YD	Da10	2016.8; 2017.5	10	YD 8
	Shaoguang	Shensuo	SS	Yuesang11	2017.6	10	SS 9
	Qingyuan	Daqiao	QD	Kangqing10	2017.9	10	QD 10
	Qingyuan	Qingcheng	QQ	283 × Kangqing 10	2018.6	10	QQ 11
	Qingyuan	Yangshan	YQ	Sha er × 109	2017.6	10	YQ12
	HunanProvince Changsha	Linmen	HCL	32 × 109	2016.3; 2017.9	10	HCL 13
YN	Zhangjiang	Nanchang	ZN	230	2017.10	10	ZN 14
	Maoming	Mingsheng	MM	Kang10	2017.10	10	MM 15
	Zhangjiang	Shuixi	ZS	Kang 10 × 230	2017.10	10	ZS 16
	Kai ping	Baihe	KB	Kangqing 10 hao	2016.11	10	KB 17
	Zhangjiang	Siyuan	ZS1	Kangqing 10 hao	2017.10	10	ZS118
	Maoming	Huazhou	MH	Xiang7920	2017.9	10	MH 19

### 2.2 PCR and sequencing

Based on previous experimental results, rDNA‐ITS was used to identify the 19 populations of Mulberry root‐knot nematode as *M. enterolobii*. Mature female (ten females for each population) was identified (the female body is white, pear‐shaped to globular, with a prominent neck of variable size) and placed into 5 μL of worm lysis buffer (WLB) containing proteinase K for DNA extraction (Williams, Schrank, & Huynh, 1992). The DNA samples were stored at −20℃ until further use. The coding region of the mtCOI gene (Large subunit ribosomal RNA gene, partial sequence) was amplified by primers LCO1490 (5′‐GGTCAACAAAT‐CTAAAGATATTGG‐3′) and HCO2198 (5′‐TAAACTTCAGGGTGACCAAAAAATCA‐3′) described by Boehme, Amendt, and Zehner (2012). The 25 μL PCR mixture contained 12.5 μL 2 × PCR buffer for KOD FX (TOYOBO), 5 μL 2 mM dNTPs, 1 μL of each primer, 2 μL of the isolated DNA, and distilled water. The PCR was carried out in a lab cycler (Applied Biosystems) under the following conditions: initial denaturation at 94°C for 4 min; followed by 35 cycles of denaturation at 94°C for 1 min, annealing at 50°C for 1 min, and elongation at 72°C for 2 min, and a final extension at 72°C for 10 min.

All amplicons were separated by electrophoresis on a 1% TBE agarose gel. The amplified products were sequenced (BGI Genomics, BGI‐Shenzhen), and the haplotypes were calculated using DNASP 5.0. The sequences obtained were submitted to GenBank and get its number.

### 2.3 Genetic diversity analysis

The sequencing chromatography was analyzed by Chromas 2.3 (https://chromas.updatestar.com/), and the DNA sequences were analyzed using the Seq Man program in DNA star 5.0 (DNA STAR, Madison USA).

#### 2.3.1 Sequence analysis

The sequence within the mtCOI gene was viewed using ClustalX 1.83. According to Librado P & Rozas J (2009), the percentage of sequence variation, nucleotide diversity (π), haplotype diversity (Hd), acid heterosis (Dxy), Fst values, Gst values, and the average number of nucleotide changes (K) of the 19 M. enterolobii populations were calculated. Gene flow (Nm) between populations was calculated using the mitochondrial‐specific gene formula Fst = 1/(1 + 2Nm) (Takahata N & Palumbi S R, 1985).

#### 2.3.2 Neutrality test

According to Kimura (1979) and Tajima (1989), the DNA fragments were subjected to Tajima's D and Fu's Fs neutrality tests at the population and group levels using DNASP 5.0 (Kimura, 1979; Tajima, 1989).

#### 2.3.3 Haplotype analysis

Base content and polymorphic loci were analyzed by MEGA 7.0 according to a previously reported method (Librado P & Rozas J, 2009). The variability between sequences was calculated based on the Kimura 2‐Parameter C K2P model, and a neighbor‐joining (NJ) phylogenetic tree was constructed using MEGA 7.0 (Tamura, Dudley, Nei, & Kumar, 2007). The haplotype network diagram was drawn by NETWORK 5.0 based on the median‐joining method.

#### 2.3.4 Molecular variation analysis

The genetic distance between populations was calculated using the MEGA 7.0 software (Fu, 2016), and the AMOVA molecular variance components and haplotype frequencies were analyzed using the Arlequin 3.5 software (Excoffier & Lischer, 2010). The correlation between genetic distance and geographic distance was calculated using SPSS (22.0).

## 3 RESULTS

### 3.1 Sequence characteristics and variation analysis of the mtCOI gene fragment in M. enterolobii

A total of 19 homologous sequences of the COI gene were amplified by PCR. After sequence comparison, 710 bp of the sequences were used for genetic research. The COI gene in *M. enterolobii* was isolated, and the GenBank number of 19 M*. enterolobii* populations is shown in the table2. A total of 25 polymorphic loci (3.5% of the total number of bases analyzed), including 12 S‐singleton sites and 13 parsimony‐informative sites, which accounted for 48% and 52% of the total polymorphisms were identified, respectively. The S‐singleton sites were located at positions 17, 188, 296, 501, 504, 587, 646, 647, 648, 650, 662, and 691 of the mtCOI fragment, and the parsimony‐informative sites were located at positions 189, 314, 328, 702, 703, 705, 706, 707, 708, 709, 710, 712, 713. The contents of a, t, c, and g were 45.79%, 28.31%, 16.60%, and 9.26%, respectively: And the content of a + t was 74.10%, showing a significant a/t bias. And the conversion/transversion rate R was 0.5.

**Table 2:** GenBank number of 19 M*. enterolobii* populations based on mtCOI gene

Order	Origin	GenBank number
1	GB	MN244944
2	HB	MN244945
3	GH	MN248512
4	YL	MN269934
5	FS	MN269935
6	GN	MN269936
7	GP	MN269937
8	YD	MN269938
9	QD	MN269939
10	QQ	MN269940
11	SS	MN269941
12	QY	MN269942
13	MH	MN269943
14	ZS	MN269944
15	ZN	MN269945
16	MM	MN269946
17	KP	MN269947
18	ZS1	MN269948
19	HCL	MN269949

### 3.2 Nucleotide and haplotype diversity analysis of mtCOI

The number of variable sites, average number of nucleotide differences (k), haplotype diversity (H), and nucleotide diversity(π) of mtCOI in *M. enterolobii* populations (Table 3) were 4.248, 0.900 (>0.5), and 0.00596 (>0.005). Tajima's D and the Fu's Fs values were −1.448795 and −1.48795, respectively, indicating that the entire population underwent neutral selection, and the changes in population were not significant. Haplotype grouping classified the 19 populations into three groups YB (Yue Bei), YN (Yue Nan), and YZ (Yue Zhong) based on the geographic distribution of *M. enterolobii* populations in Guangdong Province and the characteristics of the climatic zone. 19 M*. enterolobii* populations are grouped in Table 1. The number of haplotypes detected in the YB, YZ, and YN groups was 3, 6, and 3 (Table 3), and the Hd values of the haplotype diversity in the YB and YN groups were close, which were 0.700 and 0.733, respectively. The haplotype diversity of YZ had the highest Hd value, which was 0.952, indicating that the haplotype diversity of the three groups was rich. The nucleotide diversity of the YB and YZ groups was relatively close, being 0.00785 and 0.00708, respectively, and the nucleotide diversity of the YN group was the lowest, being 0.00355. The average number of nucleotide differences (k) is in the order of YB > YZ>YN. After testing, the values of the Tajima's D and Fu's Fs of the three groups all conform to the law of neutrality, and the group changes are not significant.

**Table 3:** Analysis of mtDNA COI gene haplotype diversity level and nucleotide polymorphism level in each group

Group	h	Hd	Π	k	Tajima's D	Fu's Fs
Total population	11	0.900	0.00596	4.248	−1.448795	−1.48795
YB	3	0.700	0.00708	5.600	−1.2142	2.535
YZ	6	0.952	0.00785	5.048	−0.9703	−1.111
YN	3	0.733	0.00355	2.533	−0.2053	1.472

### 3.3 Haplotype analyses of mtCOI in M. enterolobii

Among the 10 haplotypes identified (Table 4), Hap8 was found in seven populations with a frequency of 36.7% (YL, MM, QQ, ZS, SS, KB, YQ); Hap9 existed in three populations with a frequency of 15.7% (GP, ZN, ZS1); and Hap10 appeared in two populations (GN, GB) with a frequency of 10.5%. Other haplotypes were identified only once in the tested populations.

**Table 4:** Haplotypes identified in *M. enterolobii* populations based on COI mitochondrial DNA gene sequence

Haplotype	Haplotype sequence	*N*	Geographic distribution	Haplotype frequency
Hap1	ATCTTCCCTCTATGCTGTGACCCAT	1	HB	5.3%
Hap2	ATCATCCCACTATGCTGTGACCCAT	1	QD	5.3%
Hap3	ATCTTCCCTCTATGCGTGACAAAT	1	YD	5.3%
Hap4	CACTTCCCTCTATGTGTGACAAAT	1	FS	5.3%
Hap5	AACTTCCCTCTATGCGTGACCCAA	1	HCL	5.3%
Hap6	AACTTCCGTCTATGCGTGACCCAA	1	GH	5.3%
Hap7	AACTTTCCTCTATGCGTGACAAAT	1	KB	5.3%
Hap8	AACTTCCCTCTATGCGTGACAAAT	7	YL 、MM、QQ、 ZS 、 SS 、 KB、 YQ	36.7%
Hap9	AACTATCCTCTATGCGTGACAAAT	3	GP、 ZN 、ZS1	15.7%
Hap10	AATTTTCCTCTATGCGTGACAAAT	2	GN 、 GB	10.5%

### 3.4 Phylogenetic analysis of mtCOI haplotypes in M. enterolobii

**FIGURE 1:**
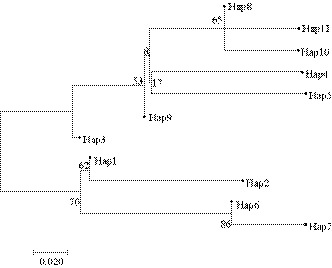
Phylogenetic tree of 10 aforementioned mtCOI haplotypes was constructed using neighbor‐joining (NJ) method under Kimura 2‐parameter model in MEGA 7.0. This shows that haplotype topology did not correlate with geography. There was a correlation between locations, and the 10 haplotypes could not be divided into single‐line groups corresponding to different geographic regions.

Phylogenetic tree of 10 aforementioned mtCOI haplotypes was constructed using neighbor‐joining (NJ) method under Kimura 2‐parameter model in MEGA 7.0 (Fig. 1). The results showed that haplotype topology did not correlate with geography. There was a correlation between locations, and the 10 haplotypes could not be divided into single‐line groups corresponding to different geographic regions.

### 3.5 Haplotype mediation network map of mtCOI

**FIGURE 2:**
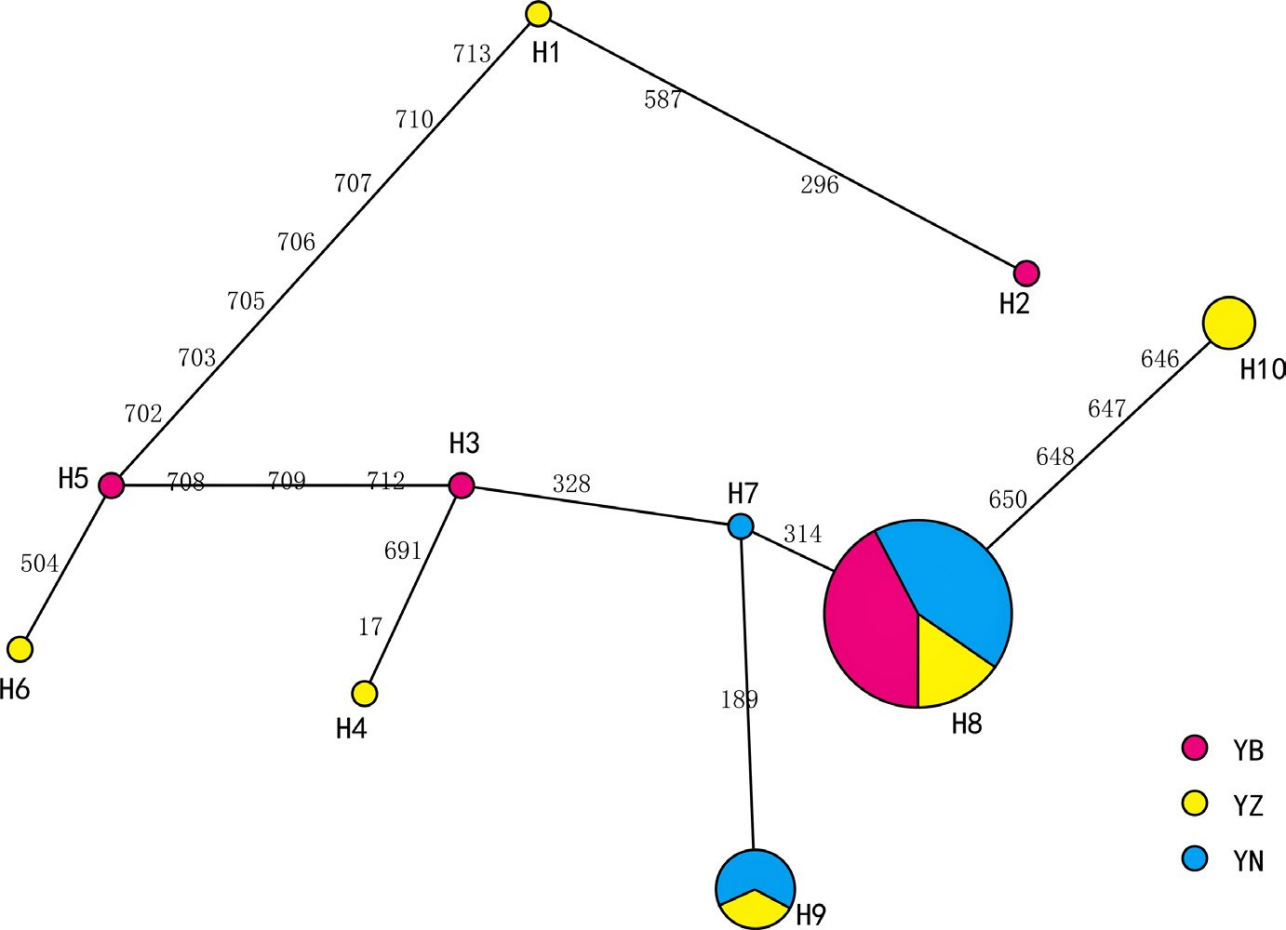
Intermediary network of *M.enterolobii* built based on mtCOI haplotypes by NETWORK 5.0 based on the median‐joining method. The results showed Hap8 was shared by seven geographic groups (YL, MM, QQ, ZS, SS, KB, YQ), Hap9 by three (GP, ZN, ZS1), and Hap10 by two (GNGB). A radial network map was formed around Hap8, in which Hap1, Hap2, Hap3, Hap4, Hap5, Hap6, and Hap7 were characteristic for the HB, QD, YD, FS, HCL, GH, and KB group, respectively. Explains the evolutionary relationships between each haplotype and the distribution of each geographical group, further supporting the phylogenetic tree.

An intermediary network of mtCOI haplotypes of the root‐knot nematode populations was then constructed. As shown in Figure 2, Hap8 was shared by seven geographic groups (YL, MM, QQ, ZS, SS, KB, YQ), Hap9 by three (GP, ZN, ZS1), and Hap10 by two (GN, GB). A radial network map was formed around Hap8, in which Hap1, Hap2, Hap3, Hap4, Hap5, Hap6, and Hap7 were characteristic for the HB, QD, YD, FS, HCL, GH, and KB group, respectively. This network could clearly explain the evolutionary relationships between each haplotype and the distribution of each geographical group, further supporting the phylogenetic tree (Fig. 2).

### 3.6 Genetic differentiation and gene flow analysis of the M. enterolobii populations based on mtCOI

The three geographic groups, YB, YN, and YZ, had a Fst value of 0.0169 (*p* < .05) and a Nm value of 7.02 (*p* > 4) (Table 5), suggesting sufficient gene exchange and low genetic differentiation. We observed the highest gene exchange rate between YB and YN (Nm = 10.17), whereas the gene exchange rate between YZ and YB was the lowest (Nm = 3.68). Kxy (sum/individual number) between YZ and TB was the highest (4.6), and that between YZ and YN was the lowest (4.0952). Therefore, YB and YN populations have more gene communication and less genetic differences.

**Table 5:** Genetic differentiation and gene flow analyses of three geographic groups of *M. enterolobii* based on mtCOI

Population 1	Population 2	Nm	Fst	Kxy
YZ	YB	3.68	0.15735	4.6000
YZ	YN	6.22	0.07442	4.0952
YB	YN	10.17	0.04688	4.2667

Note

Nm, pairwise comparisons based on gene flow; Fst, genetic variance within the subpopulation relative to the total genetic variance; Kxy, Average number of nucleotide differences

### 3.7 Genetic distance analysis based on mtCOI

The genetic distances among different M.enterolobii groups were calculated based on mtCOI sequences using MEGA 7.0 (Table 6). The results showed that the genetic distances between various groups ranged from 0.000 to 0.016. QD with ZN, KB, GN, ZS, SS, MH; GH and YQ; ZN and GN, ZS, SS, MH; YL and YQ; KB and GN, ZS, SS, MH; MM and GP; GN and ZS, SS, MH; GB and QQ, YQ; ZS and SS, MH, YQ; SS and MH, YQ; QD and ZS1 populations had the lowest genetic distance (0.000); however, the genetic distance between the Da and Si yuan population was the highest (0.016). The genetic distances between different populations varied little.

**Table 6:** Genetic distances between 19 M*. enterolobii* populations based on mtCOI using mega.7

	HB	QD	YD	FS	HCL	GH	ZN	YL	KB	MM	GN	GB	QQ	ZS	GP	SS	MH	ZS1	YQ
HB																			
QD	0.003																		
YD	0.003	0.006																	
FS	0.006	0.008	0.003																
HCL	0.001	0.004	0.004	0.007															
GH	0.003	0.006	0.006	0.008	0.001														
ZN	0.003	0.006	0.000	0.003	0.004	0.006													
YL	0.004	0.007	0.001	0.004	0.006	0.000	0.001												
KB	0.003	0.006	0.000	0.003	0.004	0.006	0.007	0.001											
MM	0.006	0.008	0.003	0.006	0.007	0.008	0.003	0.001	0.003										
GN	0.003	0.006	0.000	0.003	0.004	0.006	0.000	0.001	0.000	0.003									
GB	0.006	0.008	0.003	0.006	0.007	0.008	0.003	0.001	0.003	0.003	0.003								
QQ	0.006	0.008	0.003	0.006	0.007	0.008	0.003	0.001	0.003	0.003	0.003	0.000							
ZS	0.003	0.006	0.000	0.003	0.004	0.006	0.000	0.001	0.000	0.003	0.000	0.003	0.003						
GP	0.006	0.008	0.003	0.006	0.007	0.008	0.003	0.001	0.003	0.000	0.003	0.003	0.003	0.003					
SS	0.003	0.006	0.000	0.003	0.004	0.006	0.000	0.001	0.000	0.003	0.000	0.003	0.003	0.000	0.003				
MH	0.003	0.006	0.000	0.003	0.004	0.006	0.000	0.001	0.000	0.003	0.000	0.003	0.003	0.000	0.003	0.000			
ZS1	0.011	0.016	0.008	0.011	0.013	0.014	0.008	0.007	0.008	0.006	0.008	0.008	0.008	0.008	0.006	0.008	0.008		
YQ	0.003	0.006	0.004	0.004	0.006	0.000	0.001	0.000	0.003	0.003	0.003	0.000	0.003	0.000	0.003	0.000	0.000	0.008	

### 3.8 Correlation between geographic distance and genetic distance

**FIGURE 3:**
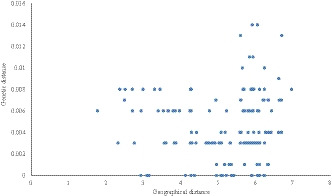
Relativity between genetic distance and geographic distance for pairs of *M. enterolobii* populations based on COI gene. The results showed that there was no significant correlation between the genetic distance and the natural logarithm (LN km) matrix (r=−0.123, p=|−0.155|>0.05) of the geographic distance among samples collected, indicating that geographical distance is not the main factor leading to root‐knot nematode population differentiation.

The correlation between genetic distance and geographic distance based on mtCOI was investigated (Fig. 3). The results showed that there was no significant correlation between the genetic distance and the natural logarithm (LN km) matrix (r = −0.123, p = |−0.155|>0.05) of the geographic distance among samples collected, indicating that geographical distance is not the main factor leading to root‐knot nematode population differentiation (Table 7).

**Table 7:** Natural logarithm of the geographic distances between 19 M*. enterolobii* populations based on mtCOI using excel

	HB	QD	YD	FS	HCL	GH	ZN	YL	KB	MM	GN	GB	QQ	ZS	GP	SS	MH	ZS1	YQ
HB																			
QD	5.976																		
YD	4.412	2.944																	
FS	6.22	5.651	6.282																
HCL	3.797	5.880	4.917	6.202															
GH	6.327	6.617	6.094	6.626	6.353														
ZN	3.563	5.695	4.772	6.243	3.303	6.343													
YL	5.926	4.276	6.113	5.587	5.838	6.697	5.917												
KB	5.192	4.947	6.110	5.802	5.081	6.432	5.223	5.337											
MM	5.913	4.248	5.078	5.807	5.796	6.698	5.913	2.493	5.334										
GN	5.7133	3.446	5.969	5.608	5.685	6.640	5.698	4.259	4.960	4.280									
GB	4.262	5.672	5.078	6.270	3.871	6.420	3.658	5.900	5.309	5.905	5.700								
QQ	3.538	5.693	4.764	6.219	3.401	6.343	2.370	5.902	5.170	5.903	5.691	3.830							
ZS	3.857	5.672	4.840	6.216	1.774	6.224	4.265	5.958	5.180	5.957	5.754	4.700	4.125						
GP	4.276	5.685	5.108	6.273	3.871	6.417	3.671	5.910	5.298	5.901	5.706	3.157	4.261	4.683					
SS	5.921	4.284	6.116	5.611	5.839	6.701	5.931	2.322	5.352	2.760	4.282	5.907	5.990	6.005	5.906				
MH	5.090	6.093	4.369	6.354	5.321	5.955	5.232	6.229	5.713	6.231	6.092	5.422	5.030	5.042	5.416	6.242			
ZS1	5.913	4.283	6.112	5.620	5.846	6.700	5.937	3.095	5.706	2.933	4.909	5.909	5.986	4.684	5.906	6.246	6.241		
YQ	3.611	3.538	4.422	5.596	4.290	6.054	5.121	6.045	5.357	4.300	5.876	5.331	4.961	5.746	6.054	6.059	6.096	4.272	6.969

### 3.9 Genetic variance of the M. enterolobii populations

Based on the AMOVA method, the Arlequin software was used to analyze the genetic variation among YB (the Yuebei group), YZ (the Yuezhong group), and YN (the Yuenan group). The intrapopulation differentiation parameter FST was 0.04498 (*p* < .0001). The variations within a population accounted for 95.5% of total variation, and the variations among populations accounted for 4.50% of total variation (Table 8). These results indicated that the genetic differentiation of the root‐knot nematode populations was mainly due to the variations within each group rather than those among different groups.

**Table 8:** Genetic variation analyses of 19 M*. enterolobii* populations based on mtCOI

Source of variation	*df*	Variance components	Percentage of total variation（%）
Between groups	4	0.01754 Va	21.05
Within a group	15	0.40603 Vb	78,96
Total variation	19	0.42358	100

## 4 CONCLUSION AND DISCUSSION

In the present study, we carried out an in‐depth investigation of the genetic diversity, population structure, as well as the association between the geographic distribution and genetic distance of *M. enterolobii* populations based on the mtCOI gene for the first time. The *M. enterolobii* populations comprised mulberry samples from 19 different main sericulture areas in Guangdong and Hunan Provinces in China. The intermediary network diagram of mtCOI haplotypes as well as the results of Tajima's D test and gene flow analysis suggested adequate gene exchange between different geographic groups, leading to low genetic diversity. In addition, we found that the genetic variation of *M. enterolobii* was mainly contributed by the variation within, rather than between different geographic groups; and there was no significant correlation between genetic distance and geographic distribution.

In recent years, molecular tools have found their applications in the research of genetic diversity, population differentiation, and evolutionary or taxonomic relationships between closely‐related species. So, the genetic diversity study of parasite populations has gained increasing interests. Mitochondrial DNA markers have emerged as a useful tool due to a relatively higher Fst value compared with nuclear sequences, and the mtDNA of nematodes was reported to evolve more quickly than that of other parasites (Anderson, Blouin, & Beech, 1998; Blouin, Yowell, & Courtney, 1995; Dantas et al., 2013). Mitochondrial DNA genes (such as mtCytb) have been widely used to resolve the phylogenetic relationships between nematodes at the subspecies, species, genus, and order levels (Liu et al., 2013; Plantard, Picard, & Valette, 2008; Zhao, Li, & Ryan, 2012). Here, the mtCOI gene was sequenced in 19 M*. enterolobii* populations to determine the genetic diversity of *M. enterolobii*. The results of this study are similar to those of Wang (2015) who used the COI gene to study the genetic diversity of soybean cyst nematode population. However, its overall fixation coefficient Fst value is 0.27442 and its overall gene flow Nm is 1.322. The results showed that there was certain genetic differentiation among various populations, but the level of gene exchange was also high. The Fst value of the total fixed coefficient and the Nm value of the total gene flow in this study are 0.0169 and 7.02, respectively. The results show that the total population has little genetic differentiation, sufficient gene exchange, and differences. It may be caused by the fact that different nematodes come from different regions. There is no correlation between genetic distance and geographical distance between the two. The results of Li, Zhang, and Tang (2015) analysis of the genetic differentiation level of the wild population of Corbicula flumina in Hongze Lake are also different. Its 15 haplotypes are clustered into two obvious branches, indicating that the population of Hongze Lake has genetic differentiation. The results of the neutrality test and the distribution map of divergence points show that the population size of Hongze Lake has remained relatively stable and has not experienced expansion. These conclusions are completely different from the results of this study. This may be caused by different species, as different species may lead to different results of genetic diversity analysis using the same primer. This research result is consistent with the study on Genetic Diversity of Heterodes Nematodes in Sichuan Based on Mitochondrial COX1 Gene by Zhu, Jian, and Wang (2015) and his team. Both research results indicate that the population has experienced expansion, but without no obvious population differentiation, and no evident geographic genetic structure has been formed. The topological structure of the phylogenetic tree shows that the phylogenetic relationship (Figure. 1) between the various groups of the root‐knot nematodes of the mulberry trees in Guangdong Province has no direct correlation with the geographical area in which they are distributed, and does not form a significant geographic genetic structure. The haplotype Hap8 is divided into three large groups. They all exist in the region, and different haplotypes from the same geographical group are not clustered, which further shows that the geographic populations of mulberry root‐knot nematodes in Guangdong province communicate more frequently and have a lower level of genetic differentiation. The haplotype network diagram (Figure. 2) shows that the haplotype Hap8 may be the origin center of the *M. enterolobii* species in Guangdong Province, and the remaining haplotypes have evolved from this. In addition, the low genetic differentiation among the populations and the haplotype relationship not conforming to the geographical distribution further indicate that the various groups of mulberry root‐knot nematodes in Guangdong may originate from a recent large population and then spread to other regions (Yu, 2009). And it suggested rapid population growth of *M. enterolobii*.

In general, standard indices of genetic diversity are represented by the number of different haplotypes, haplotype diversity (h), and nucleotide diversity (π) (Wu et al., 2009). Our results detected a high haplotype diversity (0.900 > 0.5) and a high level of nucleotide diversity (0.00596 > 0.005) of the *M. enterolobii* populations, which is a common observation in a number of other invertebrate animals with large standing population sizes and extremely high fecundity (Lavery, Moritz, & Fielder, 2008; Grant W A S & Bowen B W, 1998). This reflects the high matrilineal effective population size of *M. enterolobii* or population expansion after a period of low effective population size, as rapid population growth enhances the retention of new mutations.

In this study, the genetic diversity of *M. enterolobii* parasitic on mulberry was evaluated using mitochondrial genes. In 2010, Tigano M et al. amplified the genes using the intergenic region (IGS) of the rDNA, the cytochrome oxidase subunit II (COII), and 16S rRNA genes (mtDNA). The genetic diversity was analyzed by neutral molecular markers, namely AFLP, ISSR, and RAPD. The results showed a low level of diversity among the isolates tested, indicating that *M. enterolobii* is a genetically homogeneous root‐knot nematode species. However, the results of this study revealed sufficient gene exchange in the population, with a high genetic level. This may be caused by the fact that the host of *M. enterolobii* in this study was mulberry, which is different from previous studies. Additionally, all the 19 nematode populations were located in the south of China, and geographical factors may be another cause for the high risk of SNP. The used different genes and research methods are also possible causes for different results.

Finally, Rashidifard M et al. (2018) analyzed the genetic relationship among *M. enterolobii*, *M. incognita,* and *M. javanica* using GBS and pool‐Seq. Compared with random amplified polymorphic DNA (RAPD), restriction fragment length polymorphism (RFLP) and PCR based on rDNA, mtDNA, ITS and IGS sequences, and satellite DNA probes, this method is time‐saving and low‐cost, and does not need multiple PCR analysis. As a novel and rapid molecular genotyping tool, GBS can obtain more detailed information about genetic diversity among nematode species. In the future, it is planned to analyze the genetic diversity of *M. enterolobii* in China using GBS, so as to obtain more detailed genetic information.

## CONFLICT OF INTEREST

The authors declare no conflict of interest.

## AUTHOR CONTRIBUTION

**Hudie Shao:** Data curation (lead); Formal analysis (lead); Investigation (lead); Methodology (lead); Resources (equal); Writing‐original draft (lead); Writing‐review & editing (equal). **pan zhang:** Resources (equal); Software (equal); Writing‐review & editing (equal). **Chunping You:** Conceptualization (lead); Funding acquisition (equal); Project administration (equal); Resources (lead); Writing‐review & editing (lead). **Chuanren Li:** Conceptualization (equal); Writing‐review & editing (equal).**Yan Feng:** Resources (equal); Supervision (equal); Writing‐review & editing (equal). **Zhenwen Xie:** Funding acquisition (lead); Project administration (lead). 

## Data Availability

The assembled gene sequences of *M. enterolobii* are available in NCBI (MN244944, MN244945, MN248512m and MN269934‐MN269949, respectively). The authors affirm that all data necessary for confirming the conclusions of the article are present within the article, figures, and tables.
